# A realist evaluation of a home-based end of life care service for children and families: what works, for whom, how, in what circumstances and why?

**DOI:** 10.1186/s12904-022-00921-8

**Published:** 2022-03-08

**Authors:** Cari Malcolm, Katherine Knighting

**Affiliations:** 1 School of Health and Social Care, Edinburgh Napier University, EH11 4BN Edinburgh, Scotland, UK; 2 Faculty of Health, Social Care and Medicine, Edge Hill University, St Helens Road, Ormskirk, Lancashire L39 4QP UK

**Keywords:** End of life care, Paediatrics, Palliative care, Home care, Realist evaluation

## Abstract

**Background:**

Children’s palliative and end of life care is underpinned internationally by a commitment to provide care and support in the family’s preferred place, which may include home, hospital or hospice. Limited evidence on models of best practice for the provision of children’s end of life care at home is available. This realist evaluation of a novel, home-based end of life care service explored what works for whom, how, in what circumstances and why.

**Methods:**

Adopting principles of realist evaluation, an initial programme theory (IPT) was developed from multiple data sources including a scoping review, service documentation review, audit of service data, and qualitative data gathered from stakeholder (*n* = 6) and family interviews (*n* = 10). Three families who had used the service were identified as case studies and interviews with professionals involved in their care (*n* = 20) were conducted to test the IPT. The findings informed the revised CMOs illustrating the contexts and mechanisms which underpin how and why the service works and for whom.

**Results:**

CMO configurations were identified explaining how and why the service works for families across five core components: anticipatory approach to care planning and delivery, advance care planning, service responsiveness and flexibility, 24/7 nurse-led service with 24-h medical support, and partnership working. Key mechanisms include establishing trusting relationships, building skills and parent confidence to deliver care, early advance care planning discussions with clear documentation, providing a single point of contact for families, workforce planning and resources to deliver the service as intended, effective communication and leadership within and between providers of the service, and use of joint policies and procedures. Recommendations for future development and expansion of the service are also discussed.

**Conclusions:**

The findings highlight core components making this service a success and areas of challenge which continue to be addressed as the service develops. With increasing demand for home-based end of life care for children these components provide a structure which can help to guide service development to meet the needs of these families in other regions to ensure that children and families receive good quality care in their place of choice.

**Supplementary Information:**

The online version contains supplementary material available at 10.1186/s12904-022-00921-8.

## Background

Children’s palliative and end of life (EOL) care is underpinned by an international commitment to ensure care and support can be provided in the family’s preferred place, which may include settings such as home, hospital or hospice [1–3]. There is increasing international evidence highlighting that families wish to remain at home towards the end of their child’s life, supported by specialist palliative care professionals [4–6]. In order to provide home-based EOL care, appropriate systems and services must be in place to address the complexities around EOL care in this population and importantly, to ensure that the diverse range of complex life-shortening conditions observed in children and resultant symptom profiles are able to be managed and supported effectively and in line with the wishes of families. A recent scoping review found there is minimal published evidence on models of care providing children’s EOL care at home that could inform best practice [7]. However, the evidence suggests that key aspects of home-based care provision at the EOL include access to nursing and medical care any time of day or night, including staff with specialist knowledge and expertise in children’s palliative care, effective symptom management and partnership working between a range of disciplines and services [7].

Within Scotland, the paucity of home-based EOL care services was addressed by developing a bespoke model of care designed to support families to access specialist paediatric EOL care in their home. The aim of this evaluation, employing realist evaluation methodology [8] was to address the question ‘What are the components of home-based EOL care services that work, for whom, how, in what circumstances and why?’

## Methods

### Design and setting

Evaluation of the Care 24 Lothian service was grounded in principles of realist evaluation and reported in accordance with the RAMESES II reporting standards for realist evaluations (see Supplementary file 1) [9]. In contrast to traditional service evaluations which aim to assess the effectiveness of a service based on the extent to which its intended outcomes were achieved, realist evaluations adopt a theory-driven approach to evaluating programmes and services to better understand how they operate in practice by considering the questions, ‘what works, for whom, under what circumstances, and how’. [8, 10] Most interventions are complex in nature and comprised of several components or parts that interact with each other and with the complex social environments in which they are situated. Realist evaluation sets out to better understand how interventions operate in different social contexts and are increasingly used to evaluate complex interventions [11, 12]. The MORECare guidance statement on best practice when undertaking evaluations of EOL care further highlight the complexity of such interventions which bridge symptom relief, physical, emotional, social and spiritual care [13]. EOL care and care services, albeit in an adult population [14, 15] have been evaluated successfully using a realist approach. A realist evaluation is ideally suited to evaluate Care 24 Lothian, which is a complex service involving two distinct organisations, NHS Lothian and a children’s hospice (Children’s Hospices Across Scotland - CHAS), working in partnership to deliver coordinated and tailored EOL care and support to children and families in their home.

The Care 24 Lothian service, established in 2014, is the first, and currently only, formalised home-based EOL care service in Scotland. It serves a geographical area spanning 700 mile [2] and an estimated population of 177,000 children aged 0–16 years [16] and falls under the NHS Lothian Health Board. Key aspects of the service include:

- Shared care service with two core providers, NHS Lothian Children’s Services and CHAS, engaging in joint working and collaboration to deliver expert paediatric EOL care.
- Referrals to the service can be made for any child aged from birth to 16 years, living within the NHS Lothian boundaries, and recognised as entering the last weeks/days/hours or life.
- Service is designed to be put in place quickly and stepped down appropriately should the child’s condition stabilise or following their death.
- Each child and family referred to the service are assigned a Lead Professional who is responsible for ensuring effective coordination and provision of care. Typically, this is a member of the Community Children’s Nursing Service (CCN) but for those children with an oncology diagnosis, it would be a member of the Paediatric Oncology Outreach Nursing Service (POON). A Lead Clinician with overall responsibility for the child’s medical care is also identified and is normally a consultant from the relevant medical specialty.
- Nurse-led service providing access to direct care at any time (24 h a day and 7 days a week) the family wishes. Interventions provided during home visits or via telephone include medication and symptom management, managing complex situations and emotional support, teaching and guidance, memory making interventions, active and anticipatory management of deterioration and symptoms, and care at the time of and/or following death.
- Medical support and guidance is available from the appropriate medical team as and when required.
- Families access the service using a dedicated telephone number 24 h a day and 7 days a week. Care is delivered primarily CCN or POON service during the hours of 08:00 until 18:00 and by the children’s hospice nursing team from 18:00 until 08:00.

**Fig. 1:**
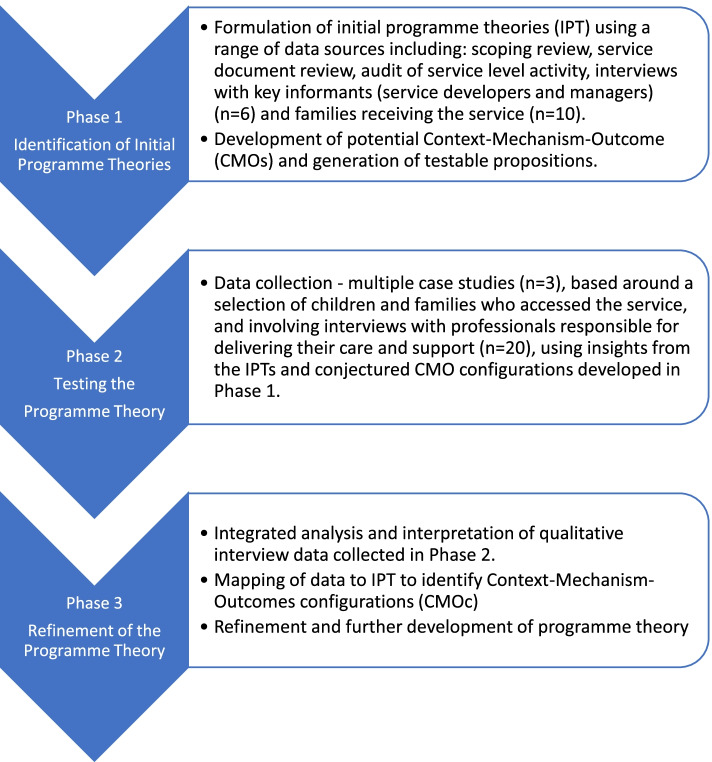
The realist evaluation process indicating the sources of data and activity included within each phase (adapted from Salter and Kothari 2014) [19]

Realist evaluations typically consist of three broad phases. The first seeks to identify how the programme or service is ‘meant’ or expected to work in practice and this is referred to as candidate or initial programme theories (IPT). Data is collected from multiple sources to accomplish this, and often includes both a review of the existing evidence base and engagement with those involved in the development and management of the service [9]. These data are then used to develop propositions or hypotheses about how the service is intended to operate. The IPT comprises propositions expressed in the form of Context-Mechanism-Outcome (CMO) configurations which examine ‘how the context and mechanisms influence the outcomes of an intervention’ (p. 201) [17]. Detailed definitions of the concepts of context, mechanism and outcome as they are understood within realist evaluation methodology are provided in Table 1. In the second phase, the IPTs are tested using empirical data that seeks to explain how the programme or service actually works in ‘real life’ contexts from the perspectives of those involved in its operation. Participants are invited to confirm, refute or refine the IPT about the service. In the third and final phase, the overall programme theory is refined through analysis and interpretation of the data to establish how in different contexts or circumstances (C), various mechanisms (M) are triggered to generate outcomes or results (O). These are communicated as CMO configurations [9]. Figure. 1 illustrates the realist evaluation process and sources of data and activity included within each phase of this evaluation.

**Table 1:** Definition of context, mechanism and outcome within realist evaluations [8, 10, 18]

**Context**	The settings or conditions within which an intervention is implemented will either enable or inhibit mechanisms from operating. Context is much broader than physical locality and can include, for example, interpersonal and social relationships, economic and political structures, programme participants, programme staffing, organisational context and cultural norms.
**Mechanism**	Mechanisms describe what enables an intervention to ‘work’ or bring about any effect (positive or negative). It is not the intervention itself that works, but a combination of the resources (for example, skills, information, support) they offer and the participants’ reasoning (for example, values, beliefs, attitudes) in response to the these.
**Outcome**	The intended or unintended effects or consequences of an intervention. They are a result of the activation of different mechanisms in different contexts.

### Participants and data collection

#### Phase 1 – identifying the programme theory

Identification of the IPT was informed by a range of data, including a scoping review, key informant interviews (service managers/developers and families), a service document review, and an audit of service level activity. The scoping review set out to establish the current evidence base relating to paediatric EOL care provided at home [7]. Specifically, the review aimed to identify and describe those models of care or services which enable EOL care to be provided at home. In addition, it explored evidence surrounding the perspectives and experiences of families receiving paediatric EOL care at home and professionals delivering this care.

Semi-structured interviews with key stakeholders (*n* = 6) involved in the design, development and management of the service were undertaken to explore their accounts of Care 24 Lothian which included: drivers informing service development; service implementation; intended delivery and outcomes; anticipated impacts on families and practice; and future development of the service. Interviews with parents of children (*n* = 10) who received care from the service were also undertaken to explore their experiences of and reflections on receiving EOL care for their child at home [20]. The document review examined strategic papers related to the development and implementation of the service including the service framework, standard operating procedures and relevant policies and standards. An audit of service activity data was undertaken to assess the extent to which the intended delivery of Care 24 Lothian, as outlined in the service framework, has been met since the service came into operation in 2014.

#### Phase 2 -testing the programme theory

A multiple case study approach [21] was adopted to test the IPT and examine the extent to which the service is working as anticipated both for families and those providing the service. A ‘case’ was defined as a family who accessed the service from the time period covered within this evaluation, which was the date of the service’s inception (November 2014) to the study’s commencement date (December 2018). Three cases were purposively sampled from the 10 families who participated in an interview during phase 1, to identify those who would best represent a diverse range of experiences and characteristics of the service (Table 2).

**Table 2:** Factors informing case study selection

Level of involvement and care provided to families by both the CHAS and NHS Lothian teams who are the core providers of the Care 24 Lothian service	
Child’s diagnosis (oncology/non-oncology diagnosis)	
Length of time family accessed and were supported by the service	
Age of child at death	
Service time point – to ensure inclusion of both families who used the service near its inception and those who used it more recently	
Place of death (home, hospice, other)	

The three cases chosen facilitated the identification and invitation of professionals for this phase of the evaluation. This included the Lead Clinician and Lead Professional for each case. Professionals external to the Care 24 Lothian service, but with significant involvement in supporting these families at the EOL, such as physiotherapists or GPs, were also invited to take part. Professionals and care providers were identified from archived case notes. Initial contact was made by a member of the evaluation team who sent an email to all relevant professionals introducing the purpose of the evaluation and providing them with an invitation to take part. Following consideration of this invitation, professionals were asked to return an ‘Agreement to Participate’ form to the evaluation team. Professionals were then contacted directly to arrange a suitable date and time for the telephone interview. Informed written consent was obtained prior to each interview.

Case study interviews were set within the context of the family’s experience and set out to test the propositions developed in phase 1. Professionals were invited to indicate the extent to which they agreed with or refuted the proposition statements and to explore accounts of their role within the service, key features of the service which supported the EOL care provided, and any challenges encountered.

### Data analysis

Adhering to a key analytical principle within realist evaluation, a retroductive and iterative approach to data analysis was adopted across each phase. Retroduction refers to “the identification of hidden causal forces that lie behind identified patterns or changes in those patterns” (p.1) [22] Using both inductive and deductive reasoning as well as the researchers’ ‘hunches’ or insights, and multiple data sources, we moved iteratively between the developing IPTs and the data to identify those mechanisms operating within a context that led to an outcome [9, 22].

#### Phase 1 – identifying the programme theory

A thematic framework approach to data analysis was adopted [23]. Key informant interviews were digitally recorded and transcribed verbatim with any identifying data removed. Transcripts of recorded interviews, the scoping review and service documents were managed using the software NVivo V.12 Pro and analysed using framework analysis [23]. Framework analysis gives a structured, standardised approach to the collection and management of qualitative data and allows for the description and interpretation of key issues arising from the data. Analysis proceeds through five stages: familiarisation; identification of a thematic framework; indexing; charting; and mapping/ interpretation. During the analysis of qualitative interview data, the team drew on what was learnt from the other sources (ie service documents, scoping review), using an approach similar to Dalkin and colleagues [24], where ‘initial hunches’ were used to develop codes relating to aims of the research and the core components of the service. A clear description of the code was documented to keep a clear record of our thinking behind the ‘hunches’ or insights and amended as they developed. This helped to maintain the link between mechanisms and outcomes, rather than coding separately by context, mechanism and outcomes which would result in more fragmented themes [24].

Following the development of a coding framework, subsequent synthesis and interpretation of the data facilitated the development of initial programme theory propositions about how the service was intended to operate. The IPT comprises these propositions, in the form of CMO configurations, to outline how, for whom and under which circumstances the Care 24 Lothian service was expected to work to ensure effective home-based EOL care for children and their families.

#### Phase 2 – testing the programme theory

Case study interviews were analysed using a thematic framework approach as described in phase 1. The framework approach was suitable to this multiple case study design as it allowed for comparison of data *across* cases as well as *within* individual cases and thus captured both unique and shared experiences. Transcripts were independently reviewed and coded by two members of the evaluation team through the two stages of this phase. In stage one, the initial coding framework was based around the aims of the research to explore each professional’s perspective on effectiveness of the service delivery, facilitators and barriers to the service and future needs of the service. This was situated within the context of the family’s engagement with and use of Care 24 Lothian. The transcripts were then examined in stage two to identify the extent to which the data concurred with or refuted the proposition statements in the IPT. The team met regularly to discuss the ongoing analysis and refinement of the programme theory.

#### Phase 3 – refining the programme theory

The final phase involved integrated analysis and interpretation of case study interview data from phase 2 to revise the IPT. This was accomplished by iteratively testing and refining the data against the original CMO statements and proposing revised explanations of how the service has worked in practice since its inception.

## Results

### Phase 1 – identifying the initial programme theory

Analysis of the data gathered during phase 1 resulted in the development of five theoretical propositions making up the IPT. These are presented in Table 3.

**Table 3:** Theoretical propositions making up the IPT

Component of the service	Propositions
1. **ANTICIPATORY APPROACH TO CARE PLANNING AND DELIVERY**	If the Care 24 Lothian service adopts an anticipatory approach to the care of children and families **(Context)**, then the service is able to plan and deliver the majority of care during regular working hours (08:00–18:00) **(Mechanism)** thus reducing the need for access to out of hours (OOH) care by families **(Outcome)**.
2. **ADVANCE CARE PLANNING**	When advance care planning that supports families’ choices and goals for quality palliative and EOL care **(Context)** underpins the service provided by the Care 24 Lothian team and is prioritised within the care delivered **(Mechanism)**, this can lead to delivery of effective and person-centred EOL care for the child and family **(Outcome)**.
3. **SERVICE RESPONSIVENESS AND FLEXIBILITY**	Access to care in different settings (home, hospice, hospital) and supporting families to move between settings according to their fluctuating needs at that time **(Context)**, by the service responding in a flexible and need-driven way **(Mechanism)**, will provide families with choice over the preferred place of EOL care and they will be supported to remain there as long as they wish **(Outcome)**.
4. **24/7 NURSE-LED SERVICE WITH 24-HOUR MEDICAL SUPPORT AVAILABLE**	Care 24 Lothian is a 24/7 nurse-led service providing home-based EOL care to children and families **(Context)**. It is delivered by nurses with appropriate knowledge and expertise in children’s palliative and EOL care and supported by 24-h access to expert medical care **(Mechanism)**. This approach leads to the provision of consistent, high quality care and support to children and families **(Outcome)**.
5. **PARTNERSHIP WORKING**	An integrated service where NHS Lothian and CHAS work in partnership to deliver home-based EOL care for children and families **(Context)** requires effective communication, leadership and adoption of joint policies, procedures and documentation **(Mechanism)** to ensure the provision of consistent, high quality care and support to families **(Outcome)** and to enhance team working **(Outcome)**.

### Phase 2 and 3– testing and refining the Programme theory

Twenty-five professionals were invited to participate in a case study interview - nine associated with case one, eight with case two and eight with case three. Twenty professionals accepted the invitation with one professional being involved in all three cases resulting in 22 interviews. Most were part of the Care 24 Lothian team or played a direct role in provision of the service. In addition, there were two GPs who whilst not part of the service team, worked in collaboration to support these families to be at home for their child’s EOL. Semi-structured interviews were conducted with the 20 participants via telephone and lasted between 19 and 68 min in length. The role of professionals included in each case study are reported in Table 4.

**Table 4:** Role and number of professionals participating in each case study

Role	Case 1 (***n*** = 9)	Case 2 (***n*** = 6)	Case 3 (***n*** = 7)
Hospice Advanced Nurse Practitioner	1	0	0
Hospice Nurse	1	1	1
Community Children’s Nurse	3^b^	2	2
Consultant Paediatric Oncologist	0	0	2*
Consultant Paediatric Neurologist	1^a^	0	0
Paediatric Oncology Outreach Nurse	0	1^b^	1^b^
Community Health Support Worker	1	0	0
Palliative Care Nurse Specialist	1	1	1
GP	1	1	0

^a^Lead Clinician; ^b^Lead Professional

A descriptive summary of the cases is not provided, to ensure families are not identifiable, but, as outlined in Table 2, the cases were chosen to represent a range of characteristics and key aspects of the service. Cases included both oncology and non-oncology diagnoses. Home was the place of death in two cases. Hospice was the place of death in case three following a period of time receiving the service at home and in response to the family’s request for care to be moved to the hospice.

**Fig. 2:**
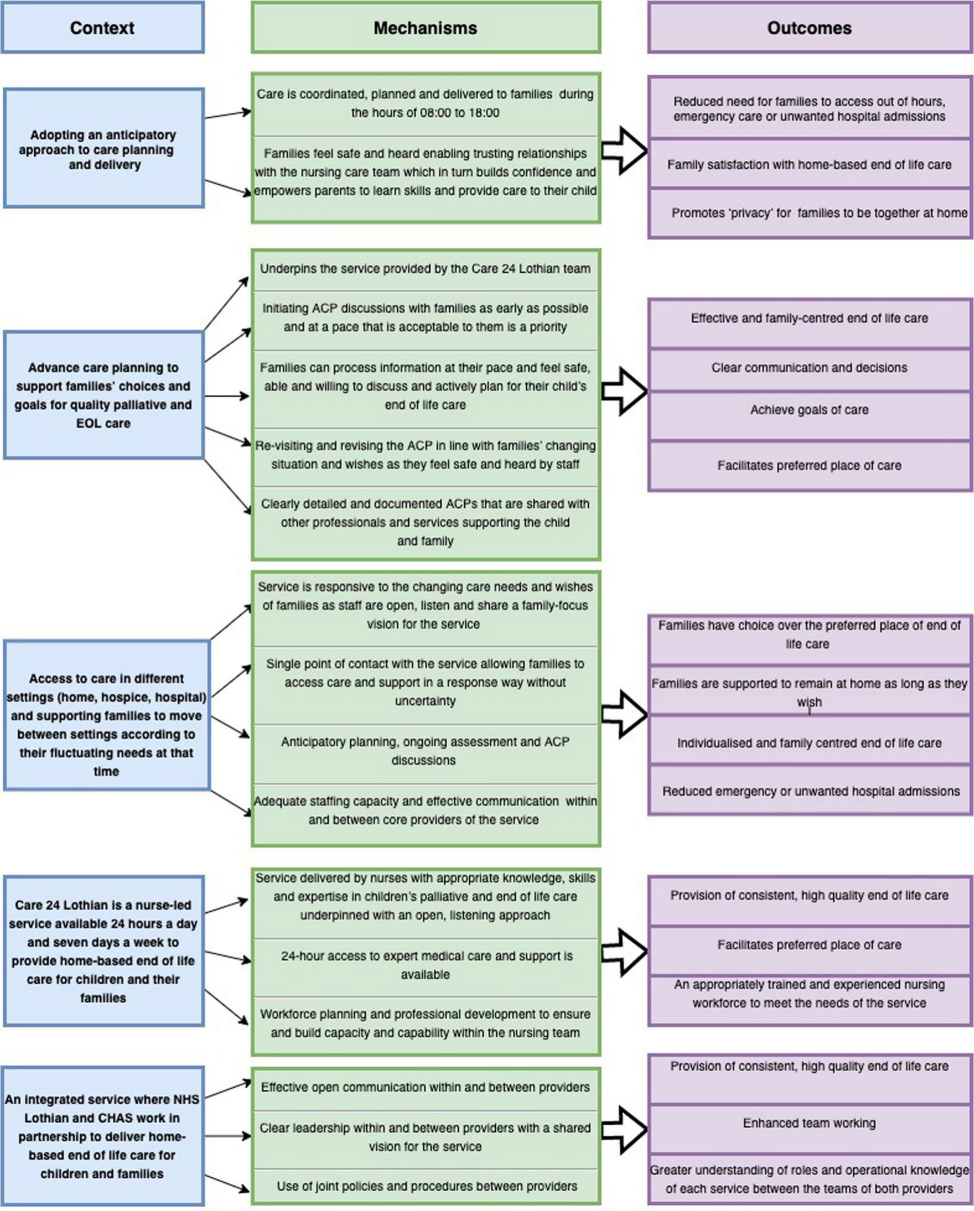
Revised and refined programme theory and CMO configurations

In phase 2, each component of the programme theory was considered to examine the level of agreement or disagreement with respect to whether the service was working as intended and to identify those facilitators and barriers to the intended service delivery. The evidence from phase 2 was then used to refine the programme theory (phase 3). The evidence enabled further explanations and greater understanding of the mechanisms and outcomes to illustrate how the Care 24 Lothian service works in practice to facilitate effective EOL care. Additionally, there are a number of suggested recommendations emanating from the case study interviews which could inform future development of home-based EOL care services. The section below presents the findings and then a summary of the refinement of the components and recommendations. The refined programme theory communicated as a set of revised CMO configurations are illustrated in Fig. 2.

### Component 1 – anticipatory approach to care planning and delivery

This component examined how the anticipatory approach to care planning and delivery was implemented in practice. Professionals, across the three cases, acknowledged that anticipatory care planning had been effective in facilitating families’ wishes to remain at home and in avoiding emergency calls to the service during out of hours:

*“I think a lot of the cases that we’ve had have shown the impact of effective assessment and anticipatory thinking when you go out to assess a child and thinking about what could happen. Your aim is to try and reduce the panic out of hours. Your aim is to try to think about what could happen in order to reduce the likelihood of families contacting out of hours because essentially that’s what you want to do.”* (Participant ID01).

From their experiences of delivering the service, professionals recognised that working closely with families during the regular hours (08:00 to 18:00) facilitated building trust and therapeutic relationships with families. It empowered families and gave them the necessary confidence to respond to their child’s care needs during out of hours. These factors, together with knowing that specialist care is available from the service 24/7 should families require it, contributed to the outcome of a reduced need to access care out of hours and allowed families the privacy to be together as a family in their home for their child’s EOL:

*“By increasing our contact and making sure that we see the patient if that’s what is needed or updates over the phone and making sure everything is in place, I think it does, and building on their confidence for them to know what to expect and what can happen, it gives them confidence then to deal with things overnight but knowing there is contact for them overnight is really helpful. So yeah, we do try and prepare them as much as possible within core hours to try and give them the confidence to deal with the maybe unexpected but for them to actually know what to expect if things were to deteriorate.”* (Participant ID22).

GPs and wider primary care services are central providers of care and support to families. Professionals delivering the Care 24 Lothian service recognised the importance of timely liaison with GP colleagues to ensure they are actively involved in the anticipatory planning and provision of home-based EOL care to families. Inclusion of GPs and ensuring they remain well informed and aware of the care needs of the child and family has been observed as an effective part of the service.

Whilst some professionals felt that less need for emergency calls or visits out of hours was an indicator of success in terms of the anticipatory approach, it was also recognised that this approach may impact on the opportunity for hospice staff, the core provider for the service between the hours of 18:00 to 08:00, to build relationships with families. It is important that families are acquainted with all aspects of the service including the team providing out of hours care, regardless of whether families go on to access such care:

*“And this is a big thing. Families need a face to face, they really value face to face contact and so that means that hopefully it’s not just a telephone number, they understand it as being a composite part of what Care 24 Lothian is, it’s a partnership.”* (Participant ID15).

Joint visits to a family’s home, by both service providers, offer a number of potential benefits. They allow members of the hospice team to play a key role in the anticipatory approach to care, provide an opportunity to meet the child and family, conduct initial assessments of their care needs and undertake a risk assessment for community lone working:

*“I think if I was the person who was called at three in the morning and happened to go out to a house to provide end of life care, to have never met that family, or the parents is challenging. Whereas I think if you’d met them before, even if you haven’t met the child I think if you’ve met the parents or the family or caregivers, I think for them and for us as professionals I think that could be quite beneficial.”* (Participant ID09).

*“When we’re giving a lot of advice over the phone I don’t know if there’s sometimes a slight inclination, whether you mean to or not, to sort of advise slightly more cautiously when you’ve never met the child or family, because actually you’re kind of advising a little bit blindly and I don’t know if you advise slightly more on the cautious side if that makes sense.”* (Participant ID09).

*“Some families I suppose aren’t asking as many questions as that and it can sometimes be difficult to get them to have the conversations. So sometimes why they end up using out of hours is more because they haven’t been as well prepared as other parents. I think there is a correlation, you know, we have seen correlations where if families just don’t want to have a conversation, they want to be at home but when you try and talk about ‘well we need to talk about what would happen and what to do if certain things happen’ and they just won’t have that conversation. Those are the ones that we’ve seen come in through, normally through ambulance into A&E and I’m just thinking about other ones as well.”* (Participant ID12).

### Summary of component 1 and recommendations

The CMO proposition being tested was ‘If the Care 24 Lothian service adopts an anticipatory approach to the care of children and families **(Context)**, then the service is able to plan and deliver the majority of care during regular working hours (08:00-18:00) **(Mechanism)** thus reducing the need for access to out of hours (OOH) care by families **(Outcome)**’. Testing this proposition against the interview and case study data, revealed that, as well as aiming to coordinate, plan and deliver all required care during regular working hours, a number of additional mechanisms were responsible for producing the desired outcomes of reducing the need for families to access Care 24 Lothian or other services out of hours and supporting families to remain at home for their child’s EOL. The mechanism of families having the opportunity to build trusting relationships with the Care 24 Lothian team facilitated their trust in the service and an openness to learning of key skills such as symptom management which then empowered families to have the confidence to care for their child out of hours and support them in being able to remain at home. Whilst this aspect of the service is working as intended, it was acknowledged that the lack of a formed relationship between families and the hospice staff does not facilitate some of these internal mechanisms and had an impact on the provision of out of hours care when it is needed. To further facilitate this component of the service it was proposed that providers from *both* parts of the service, NHS Lothian and CHAS, need to be visible to and engage with families during regular hours. It is also recommended that GPs are engaged with as early as possible so they are informed and able to support families, including out of hours when needed.

### Component 2 – advance care planning

Advance care planning (ACP) is an essential part of EOL care planning for all services and professions involved in the support of children with life-shortening conditions and their families. ACPs underpin EOL care provided by the service and therefore having an ACP in place was a pre-requisite for Care 24 Lothian. If the ACP process had not been initiated prior to the service being put in place, it was a priority for service providers to engage in conversations with families and document their essential goals and wishes around EOL care. ACP was described by professionals as being individualised to each child and family.

*“I think they’re all so unique and different and individualised that until you actually start doing it with a family…they’re all so different, the anticipatory care plans, even though they’re the same format they’re always a totally different journey for all of them I think.”* (Participant ID22).

Professionals acknowledged the importance of adopting a relaxed approach to engaging in ACP discussions with families. They described how engaging in such conversations at a pace that suits each family, often with discussions being repeated multiple times, was deemed to be most effective. Ensuring families have a clear understanding that any wishes and decisions regarding EOL care can be re-visited and amended at any time is a key facilitator to advance care planning:

*“I think, you know, recording the family’s wishes and understanding that it’s not absolute as well, you know, these conversations are ongoing so once you’ve done an ACP, it’s not written in stone, it can be changed at any time.”* (Participant ID07).

An effective way of engaging in ACP discussions with families, as described by professionals, was the ‘*drip, drip approach*’ or by being ‘*seed sowers*’ where over time, after repeated conversations and through developing increasing trust between families and the Care 24 Lothian team, families’ wishes are documented within a care plan:

*“They obviously need time, they need space and it’s very much a drip, drip approach when you’re talking about advance care planning.”* (Participant ID06).

Having up-to-date and clearly detailed ACPs is an effective tool to refer to when providing care in partnership with other services and professionals as explained by one participant:

*“So it [ACP] then gave us a very good…gave us a very quick level of trust because [GP] could realise that I wasn’t just plucking this out of the sky, that absolutely everything that I was telling him was documented in black and white in the ACP.”* (Participant ID03).

### Summary of component 2 and recommendations

The CMO proposition being tested was ‘When advance care planning that supports families’ choices and goals for quality palliative and EOL care **(Context)** underpins the service provided by the Care 24 Lothian team and is prioritised within the care delivered **(Mechanism)**, this can lead to delivery of effective and person-centred EOL care for the child and family **(Outcome)**’. The case study data supports that this proposition was being met as intended in cases where parents are able and wanting to engage in conversations about EOL care and dying with the Care 24 Lothian staff. Advance care planning is a fundamental aspect of the service and aims to ensure effective and family centred EOL care is provided. Effective ACP discussions require ongoing conversations with families that proceed at a pace appropriate for them. These facilitating mechanisms of relaxed and re-visited advance planning conversations allow families to process information at a pace they feel comfortable with, which in turn supports them to feel safe and able to make informed decisions regarding EOL care. It also ensures they have a clear understanding that their decisions are not fixed and can be revised as required due to feeling heard and their wishes valued by staff. The success of advance care planning relies on parents being in a position where they are able and willing to engage in what can be difficult discussions. An additional mechanism to facilitate advance care planning would be to involve families in these conversations as early as possible, and before the service in initiated. However, it is important to remain cognisant that all families are unique and require a flexible and tailored approach which empowers them to make and share their decisions in these nuanced conversations and takes into consideration cultural, spiritual and religious wishes.

### Component 3 – service responsiveness and flexibility

Professionals shared their perspectives on ways in which the service contributed to effective care and support of children and families. Across the three cases explored, key features of the service recognised by participants was the ‘*single point of contact*’, ability to be ‘*responsive*’ and its ‘*specialist*’ nature as communicated in a quote from one professional:

*“I think what definitely stands out as a key feature of this service is the fact that the family have one contact number during the day and then one contact number during the night. I think the family would probably say that the contact was very responsive to any identified needs when they did phone that telephone number which I think it obviously a huge thing for families to know that when they do contact they’re going to get the specialist knowledge that they want. The whole point of Care 24 Lothian is it’s like a one-stop shop. So, it’s being able to be responsive to have the right level of specialised and advanced knowledge, and to ensure that you can actually provide a face to face contact, be that nursing and also with our medical colleagues as well.”* (Participant ID06).

Participants deemed the service to be successful in offering families choice over preferred place of care at their child’s EOL. Care 24 Lothian supported families to remain at home for as long as they wanted and helped to avoid unwanted emergency admissions to hospital. The following quotes illustrate the achievement of this outcome:

*“I think probably the most helpful thing to the family was at the very end where they didn’t have to rush into hospital and [child] was able to stay at home and I think that’s where Care 24 Lothian was extremely helpful.”* (Participant ID04).

*“To me that was quite positive because the whole aim of palliative care is to support preferred place of care, and even though [family] wanted their child to die in the hospice, they wanted to be at home as long as possible and to make that happen was really through Care 24 Lothian.”* (Participant ID01).

The service, over time, has evolved to enable families to move between care settings towards the EOL in line with their choices and care requirements. Choosing to be at home for their child’s EOL care is a dynamic decision which can be revisited, and families may change their mind and wish to be cared for in an alternative setting:

*“I think it’s just about letting families know that they do have choices and empowering them to not just fit into any one box, that it can be fluid as well.”* (Participant ID20).

A responsive and flexible service is key to providing individualised EOL care to children and families. As one participant explains in the quote below, Care 24 Lothian provides family centred care by being able to adapt to the family’s changing needs and wishes as they arise:

*“It has to be flexible. It has to be dependent on the needs of that child and family and they’re all very different, all very unique. No anticipatory care plan is the same, no symptom management plan is the same…nothing is set in stone within the anticipatory care plan and I think we’ve had a few cases where things have changed [resulting in transfer from home to a different setting] and I think that just shows the flexibility and how the child and family centred it is.”* (Participant ID01).

Ensuring there is open communication and ongoing discussions with families to inform them of the choices available for place of death and supporting them through their decision making is a key facilitator. In order for the service to be in a position where changes in place of care can be facilitated for families, anticipatory assessment and planning is key. Whilst the service aims to support families’ choices regarding place of care, timing of transfer between settings is crucial as is ensuring the service has resources in place to facilitate transfers. This is reliant on effective communication pathways and planning between services.

*“[Children moving between care settings] has brought up new challenges that we hadn’t seen before, communication being the biggest one because it’s different services and we all need to know what’s happening.”* (Participant ID12).

### Summary of component 3 and recommendations

The CMO proposition being tested was ‘Access to care in different settings (home, hospice, hospital) and supporting families to move between settings according to their fluctuating needs at that time **(Context)**, by the service responding in a flexible and need-driven way **(Mechanism)**, will provide families with choice over their preferred place of EOL care and they will be supported to remain there as long as they wish **(Outcome)**. The dedicated phone number provided acts as a ‘single point of contact’ for families to access the service and specialist EOL care operates as key mechanism to avoid uncertainty and ensure care that is responsive. Moreover, being flexible and responsive to families’ care and support needs by facilitating transfer between settings at the EOL as and when required is an inherent mechanism within the service which has continued to evolve and was recognised as supporting a positive outcome of choice for families. This mechanism of the service is underpinned by core mechanism of families feeling heard and safe to share their wishes as outlined in earlier components. Anticipatory planning and ongoing assessment and discussions with families by the Care 24 Lothian staff was considered a key facilitating mechanism to ensure timely transfer between care settings so the child and family remain in their preferred place at the EOL. This outcome is also dependent on adequate staffing capacity and effective communication between services.

### Component 4–24/7 nurse-led service with 24-h medical support available

Professionals described Care 24 Lothian as a nurse-led service, delivered primarily by nursing staff with expertise in children’s palliative care, providing families with quality home-based EOL care and support:

*“It’s not just about going out to the home and putting that syringe driver up or sorting out medication, a lot of it is very much care of the child, care of the family, you know, it’s very much a nursing service I’d say, and I think we do it very well.”* (Participant ID02).

Ongoing workforce planning is important to ensure capacity and capability within the nursing team continues to grow and the service remains able to deliver the specialist care required by families. For example, there is a requirement to ensure there are sufficient nurses within the team with prescribing qualifications and advanced practice roles. One approach to building capacity within the Care 24 Lothian team involves, where possible, ensuring two nurses attend visits to a family home. Having a less experienced nurse accompany a senior nurse within the team, in parallel with other continued professional development, has proved of value in developing nursing competence and expertise in delivering community-based palliative and EOL care. Moreover, there are added benefits of this ‘working in pairs’ approach. It helps to ensure the personal safety of staff by avoiding lone working within the community. Working in pairs also addresses the highly emotional demands of this role by both allowing nurses to provide mutual support to each other. Having two nurses to manage the care and support needs of the child and wider family was beneficial in a number of situations. An example provided within the case studies was a situation where one staff member was able to attend to the care needs of the child or perhaps her/his siblings and allow the other nurse to spend uninterrupted and focus time on the emotion needs of parents and engaging in what can be difficult conversations.

Participants identified having capacity to provide care and support 24 h a day, 7 days a week as an essential component of the service as families rely on this to enable them to remain at home. They acknowledged that the service had been effective in responding to families’ needs on a 24/7 basis, however, they did identify a requirement to further improve the systems and structures in place to ensure provision of care at any time of day or night. Despite the proposed success of anticipatory approaches to care planning, as evidenced by case study interview data and the audit of service level activity undertaken in phase 1 indicating the frequency of out of hours support required is generally low, the service must maintain a capacity to respond to families at any time. Participants reported this to be a greater challenge between the hours of 18:00 to 08:00 when the hospice care team are covering the service. There were several contributory factors identified, including the intermittent and unpredictable nature of referrals to the service where there can be relatively lengthy periods without a case followed by a number of cases in succession. Additionally, the hospice, unlike the CCN team, require the capacity to cover Care 24 Lothian care overnight as well as providing palliative and EOL services to families they support in other geographical areas across Scotland.

Together with nursing care, the service ensures 24-h access to medical support. Similarly, there was agreement amongst professionals that the service has 24-h access to expert medical care. However, the findings indicate that access to expert medical care can be variable and pose challenges at times, especially during out of hours. During the hours of 08:00–18:00, there are a number of clinicians who can be called upon including the child’s medical consultant, paediatric palliative care specialists, advanced nurse practitioners and the child’s GP. When there is a need to contact medics out with these hours, it can be more problematic as there is a greater reliance on on-call cover. If the hospice’s paediatric palliative care clinicians are providing on-call cover, again, they have responsibility for covering wider community areas as well as in-hospice medical support. Whilst remote approaches such as video conferencing have proved beneficial, given the clinical complexity of many of these children and the very specialist nature of paediatric palliative care, clinicians are not always agreeable to symptom management and treatment without having undertaken a face to face assessment of the child. This is particularly the case in situations where the clinician has little previous knowledge of the child and family.

On occasions when the hospice medical team does not have capacity to respond to an out of hours call out, the service can access medical support from NHS24, a national telehealth and telecare organisation in Scotland which provides urgent care advice during out of hours. One professional spoke of accessing care via NHS24 and described how the GP on call may not necessarily have the unique skill set and experience required in paediatric EOL care:

*“I’ve been to GP meetings where we’ve been discussing Care 24 Lothian and GPs have been quite open about the fact that it’s not their area of expertise and some GPs aren’t willing to get involved because they just don’t feel confident….I guess that’s where it falls back to the fact that you’re phoning a GP who doesn’t necessarily know the family at all and who aren’t confident with syringe drivers and the medications that we’re using for the children. So I think it’s difficult kind of getting that appropriate medical cover.”* (Participant ID23).

The Care 24 Lothian team, who regularly care for children at the EOL, are competent in the pharmacological management of these symptoms and it is important that they work alongside clinicians in other specialities to ensure holistic management of the child’s care and to learn from one another. Participants recognised the key role that GPs within primary care could play in supporting the service and explained the importance of continuing to engage with and involve GPs on a more consistent basis to build capacity and address this barrier. They noted that it is often the GP who is the closest medical contact for a family geographically so there is merit in working in partnership with them.

### Summary of component 4 and recommendations

The CMO proposition being tested was ‘Care 24 Lothian is a 24/7 nurse-led service providing home-based EOL care to children and families **(Context)**. It is delivered by nurses with appropriate knowledge and expertise in children’s palliative care and supported by 24-h access to expert medical care **(Mechanism)**. This approach leads to the provision of consistent, high quality care and support to children and families **(Outcome)**. Testing this proposition against the case study data revealed that the outcome of providing consistent, high quality EOL care and support to families has been achieved but there are areas for improvement to ensure that the service can adequately maintain its intention and functioning mechanisms, particularly at time when the service is under pressure due to high concurrent demand and to be responsive to the growing number of children who may be referred as awareness of the service increases. To ensure successful outcomes continue to be achieved in a safe and consistent way, the service should identify additional opportunities and resources to support and expand existing mechanisms through workforce planning to strengthen the capacity and capability to offer 24/7 access to nursing care and specialist paediatric palliative care medical support as an outcome for all. Joint working to build capacity and confidence in GPs to provide EOL care, is recommended, particularly prescribing and symptom management, when other medics are not available to conduct home visits. There is merit in exploring the use of technology to have video calls as additional out of hours support between staff and families, and between service providers.

### Component 5 – partnership working

This component reported on partnership working between the two core providers of the service and how it has evolved and unfolded in practice. Participants reported a number of challenges that arose as a result of two distinct organisations coming together to design, develop and subsequently deliver an integrated service and acknowledged the progress in partnership and integrated working that has taken place since the service came into operation. As explained by one participant, developing a partnership to deliver an integrated service is an ongoing process:

*“I think it’s evolving. I think when I take myself back to the very early days of Care 24 Lothian, it definitely very much was or felt like a…a bit like an ‘us and them’, you know…But I think our partnership working has evolved. It takes time. It takes commitment. It takes mutual respect for services, you know. And I think in the early days they didn’t know what we did, and we didn’t really understand what they did. And I think it has evolved. I definitely feel, in particular with [child’s name], that partnership working was amazing. And …that has trickled on to lots of different cases.”* (Participant ID03).

Ongoing and effective communication between agencies delivering the service to families is essential. Participants described how there are set handover periods during the morning and early evening when there is a change in care provider from the CCN service to the hospice care team and vice versa. There is also a handover, in SBAR (Situation-Background-Assessment-Recommendation) format, provided by the Care 24 Lothian nurse who has been responsible for a child’s care over the day, which is circulated to all relevant professionals and services who may be contacted during the evening and overnight. It is important to ensure that the key individuals involved in the care of the child and family are aware of the present situation, any activity that occurred over the past 12 h as well as ongoing recommendations for the child and family. This might include, for example, the hospice care team, GP, medical clinician on-call, and other core staff.

Whilst the communication processes and procedures currently in place are largely an effective mechanism in achieving the outcome of providing consistent, high quality care and support to families and enhancing team working, communication was recognised as one of the greatest challenges in providing an integrated service like Care 24 Lothian. Participants reiterated the requirement for a continued focus on further enhancing communication processes:

*“Communication is the biggest challenge particularly when you’re working across two different areas, a health board and a charity. It’s so important to make sure that communication is streamlined. I think we’ve worked quite hard to do that. There’s still teething issues but making sure that what we have in terms of operational procedures is the equivalent to what they have, so there needs to be that understanding of what each other’s roles are within that.”* (Participant ID01).

An additional challenge was identified around ensuring effective communication between service providers in terms of the current use of different documentation for care plans and medication records and separate organisational email systems. Participants reported that this should be a priority going forward to ensure a more efficient means of communicating key information.

Participants representing both core providers spoke about how having clear insight into each other’s roles and responsibilities within their respective organisations would foster enhanced partnership working when delivering the Care 24 Lothian service. They explained the difficulty in truly getting to know each other when working largely in parallel to deliver the service. Participants suggested a number of ways in which partnership working could be enhanced including maximising opportunities for colleagues to deliver the Care 24 Lothian service together, such as undertaking joint visits to the homes of children and families receiving the service; the creation of joint posts or rotational secondments between the hospice and CCN service; and holding ‘away days’ to engage in team building activities. Clear leadership from senior managers of both organisations was seen as key to continue developing the partnership between the organisations by facilitating and supporting these types of activities and supporting mechanisms for improving communication such as using one secure email system and developing appropriate documentation to be shared and used consistently.

### Summary of component 5 and recommendations

The CMO proposition being tested was ‘An integrated service where NHS Lothian and CHAS work in partnership to deliver home-based care for children and families **(Context)** requires effective communication, leadership and adoption of joint policies, procedures and documentation **(Mechanism)** to ensure the provision of consistent, high quality care and support to families **(Outcome)** and to enhance team working **(Outcome)**. The case study data supports that this proposition was being met with the service operating as intended with respect to partnership working. Effective communication and clear leadership of both teams are important mechanisms which can be further improved along with consistent service documentation, to meet the outcomes of providing consistent, high quality care and support to families and to enhance team working. The use of approaches such as the creation of joint posts or rotational secondments between the hospice and CCN service or combined learning and development sessions may facilitate teamwork with a deeper understanding of the operational knowledge and parameters of each team. Development and use of joint documentation between both providers and use of the same secure email system is recommended.

The evidence from phase 2 has highlighted a range of individual reasoning and service level resource mechanisms to provide further explanation and understanding of how the Care 24 Lothian service works in practice to facilitate effective EOL care. As outlined in the component summaries, many of the service resource mechanisms are driven by individual reasoning mechanisms such as families being able to process information at a speed appropriate for them, feeling safe and heard by professionals, and being able to change or reconsider any decisions they have made. Professionals have also illustrated that remaining open, using good listening skills, having a shared vision which is family and child-focus, and clear communication with both families and other professionals are key to supporting the service level resource mechanisms and providing effective EOL care which meets families’ needs. The revised CMO configurations are outlined in Fig. 2.

## Discussion

This realist evaluation identified five components of an effective home-based EOL care service: anticipatory approach to care planning and delivery; advance care planning; service responsiveness and flexibility; 24/7 availability of nurse-led care with medical input as required; and partnership working. The initial programme theory was revised following the testing phase and key recommendations identified to build on the successes of the Care 24 model for home-based EOL care for children.

The development of trusting, open and supportive relationships with the nursing care team was a key mechanism which facilitated the outcome of families being satisfied with the EOL care service and a reduced requirement to access out of hours or emergency care. As families get to know and trust the care team around them, this in turn builds confidence and empowers parents to learn vital skills and provide care for their child at the EOL.

Advance care planning is an essential part of EOL care preparations, and thus ACP discussions and documentation is initiated by the service, if not already in place when families are referred. This approach to beginning, or continuing, conversations with families is a strength of the service which all participants viewed as a pre-requisite for future care provided by the service. It was recognised that these discussions are contingent on families being ready to have them and that a relaxed and individualised approach is needed. These are all mechanisms which are integral to the outcome of facilitating preferred place of care and achieving goals of care. This concurs with the findings from a recent systematic review of family experiences of receiving palliative care at home where open communication with health care professionals that was individualised to the specific needs of each family was highlighted as a priority [4]. It also aligns with evidence from the adult population which suggests that trusting and empathic relationships between patients and professionals enable empowerment to engage in advance care planning [25] and that open communication strategies, like advance care planning, with a ‘triad of experts’ (patient, family/carer, and professionals with expertise in palliative care) are effective in achieving the desired outcome of death in one’s usual place of residence [15]. However, it is important to appreciate the wider complexities and challenges around advance care planning. Whilst the use of ACPs was effective within the context of delivering home-based EOL care by this particular service, it may not be generalisable across different settings and programmes. Care 24 Lothian provides a responsive and flexible service to ensure continued choice and individualised EOL care to children and families. This component of the service has evolved to enable families to move between care settings towards the EOL in line with their wishes and care requirements and ultimately achieving the desired outcome of supporting families to remain at home for as long as they choose. Grossoehme and colleagues [26] recognise that families’ goals with respect to place of care may change in line with their child’s changing illness or their wishes. Their findings support the notion that “patients benefit from flexible, longitudinal, home-based support in programs equipped to deliver high quality EOL care” (p.155) [26]. Moreover, achieving death within a home setting, is not a necessarily a measure of the quality of EOL care but rather families having the opportunity to plan location of death and then be able to accomplish that plan, may be a more relevant outcome [27].

A central component of Care 24 Lothian was provision of specialist nurse-led care with additional medical support available at any time of the day or night as required by families. Provision of a service delivered by nurses with the appropriate knowledge, skills and expertise in children’s palliative and EOL care with 24-h access to expert medical care and support were key mechanisms and support the NICE guidance which calls for this level of care [1].

The feasibility of Care 24 Lothian is partly related to the presence of two ‘base’ sites – the Royal Hospital for Sick Children and Rachel House children’s hospice. From these bases, nursing and medical care can be provided 24 h a day and seven days a week to families living within the Lothian area. Care 24 Lothian’s successful implementation was dependent on access to a number of key resources including the CCN and POON service, Clinical Nurse Specialist for Children and Young People’s Palliative Care, Rachel House medical, nursing and care staff, and lead medical consultants from the Royal Hospital of Sick Children being within the region. Two distinct organisations coming together to design, develop and subsequently deliver an integrated service is not without challenges. The Care 24 Lothian service has successfully confronted these challenges and continue to enhance both service delivery and their partnership working. Whilst additional improvement would be of benefit, particularly around reviewing current documentation use to improve standardisation and ensure that it is fit for purpose, the service has responded to the recommendations from the evaluation and been proactive in seeking solutions and better ways of delivering care.

There are limitations which are important to consider. The evaluation is based on one model of home-based EOL care delivered in a single Health Board area within Scotland which limits generalisability and application of findings to other settings. However, palliative and EOL care services generally lack standardisation across Scotland and the wider UK resulting in inconsistencies and a lack of equitable access for families [28–30]. Moreover, it is a similar picture in other countries, such as the US, where there are few standard models for home-based EOL care due to significant variations in resources, structure of services and regulations across different geographic areas [31–33]. Yet there remains a drive by policy makers and commissioners across the UK and beyond [1–3] to ensure EOL care and death occurs in a child and family’s preferred setting, be that home, hospice or hospital. In order to empower families to set goals of care around preferred settings, effective services that are able to provide EOL care must be in place. There has been an observed expansion of both community-based paediatric palliative care services and hospital-based paediatric palliative care teams which reflects this commitment to provide home-based EOL care [5]. Further research is needed to explore how families are best empowered to have these nuanced and layered conversations and how cultural, spiritual and religious beliefs are incorporated into their decisions and their subsequent EOL care. A strength of this work was the adoption of principles of realist evaluation, a recognised and advancing methodology which is theory-driven and offers a valuable approach to understanding how a complex intervention works within specific contexts and why [10]. The authors acknowledge that the inclusion of middle-range theories could have strengthened the transferability of our findings [34]. Despite this, the findings of this realist evaluation do make a novel and much needed contribution to the evidence base. The principles and theory of effective EOL care at home can now be adopted by others to inform the development of EOL care services in response to the growing demand for care in a family’s preferred setting.

## Conclusions

Provision of EOL care in the preferred setting of children, young people and their families is a priority within palliative care policy and practice. The Care 24 Lothian service is an effective and valued model for providing high quality EOL care and support which is family oriented and led. This realist evaluation provides valuable insights into the essential components of a home-based EOL care service for children and their families which will be of particular interest to policy makers, researchers and professionals across health, social care and the third sector. The programme theory can be tested in other areas with the aim of enhancing paediatric EOL care for families who wish for their child to be cared for and die at home.

## Supplementary Information

**Additional file 1.** Supplementary File 1 - RAMESES II reporting standards for realist evaluations.

## Data Availability

The datasets generated and/or analysed during the current study are not publicly available due to ethical considerations (to ensure data confidentiality and protect the anonymity of the research participants) but are available from the corresponding authors on reasonable request.
